# What have we learned about risk assessment and interventions to prevent work-related musculoskeletal disorders and support work participation?

**DOI:** 10.5271/sjweh.4172

**Published:** 2024-07-01

**Authors:** P Paul FM Kuijer, Sten van der Wilk, Bradley Evanoff, Eira Viikari-Juntura, Pieter Coenen

**Affiliations:** 1 Amsterdam UMC, Department of Public and Occupational Health, Amsterdam, The Netherlands; 2 Amsterdam Public Health, Societal Participation & Health, Quality of Care, Amsterdam, The Netherlands; 3 Amsterdam Movement Sciences, Musculoskeletal Health, Sports, Amsterdam, The Netherlands; 4 Washington University School of Medicine, St. Louis, MO, United States; 5 Finnish Institute of Occupational Health, Helsinki, Finland.

**Keywords:** musculoskeletal disease, occupational exposure, risk assessment

## Abstract

**Objective:**

The *Scandinavian Journal of Work, Environment & Health* (SJWEH) was established half a century ago. This paper provides an overview of research on musculoskeletal disorders (MSD) published over these 50 years. Three themes are described: risk assessment, interventions to prevent work-related MSD, and interventions to support work participation. Finally, implications for future research are highlighted.

**Methods:**

A systematic literature search was performed for all papers on MSD published in SJWEH. Each paper was coded on several criteria including research topic, type of MSD, risk factor(s), and number of citations. Findings were tabulated, and discussions within the author team defined the main results and future research directions.

**Results:**

The search resulted in 1056 papers, of which 474 were included. The most reported-on MSD was low-back pain (LBP, 18%) and the most reported-on work-related risk factors were physically demanding work (14%) and psychosocial factors (12%). Research has contributed to improving case definitions, refining work-related exposure criteria, and recognizing the varying importance of physical and psychosocial factors across different MSD. Research on the association between work-related risk factors and LBP continues to emerge. Effective interventions for prevention of MSD are characterised by sufficient exposure reduction, while supporting work participation requires integrating health care, with multidisciplinary actions directed at factors involving the worker, employer, and workplace.

**Conclusion:**

Research has provided valuable insights into risk assessment, interventions for preventing work-related MSD, and supporting work participation. Intervention studies remain warranted and new areas include adopting whole-system approaches to prevent work-related MSD and promoting the concept of musculoskeletal health.

*‘The X-ray findings of the lumbar spine in the preemployment examination of 807 lumbermen are presented. From the applicants 11.4% were rejected because of roentgenological and/or clinical low back abnormalities. The youngest age group showed a high frequency of spondylolisthesis (8%). The possible role of heavy work in adolescence in the etiology of spondylolisthesis is discussed.’* (1). This text is from the abstract of the first paper on musculoskeletal disorders (MSD) in the Scandinavian Journal of Work, Environment & Health (SJWEH). The paper was published in March 1975 and described the results of a pre-employment examination of Finnish lumberman based on x-ray findings. This paper touched on three important themes of MSD research: assessment of work-related risk factors for MSD, interventions to prevent work-related MSD, and interventions to support work participation among workers with MSD. These three MSD research themes have been consistently addressed in the Journal over the past 50 years.

In 2017, van der Beek and colleagues published a research framework in SJWEH for the development, evaluation, and implementation of interventions for preventing work-related MSD (2). In this framework, risk assessment involves three steps: assessing the incidence and severity of the MSD (step 1), determining associated risk factors (step 2), and studying the underlying mechanisms (step 3). The first review paper (3) and the first supplement (4) on MSD in SJWEH, both published in 1979, anticipated these three steps in their discussions of risk assessment. Knowledge from the first three steps of van der Beek and colleagues' model leads to the next steps in the framework: development of interventions to prevent work-related MSD (step 4) and evaluation of these interventions on their (cost-)effectiveness (step 5). The first paper in SJWEH taking all five steps in assessing the effectiveness of preventive interventions for work-related MSD was published in 1978 (5). The paper described the marked increase in the prevalence and severity of vibration-induced white fingers among lumberjacks in Finland in the mid 1960s, followed by a marked decrease in the early 1970s. The authors attributed the initial increase of MSD to the use of second generation chain saws and the decrease of MSD to the use of anti-vibration saws. The last and 6^th^ step in the framework by van der Beek and colleagues is the implementation and scale-up of proven (cost-)effective interventions for prevention of MSD. However, it took until 1988 for the first paper on this topic to be published in the Journal, with a paper on implementation of ergonomics measures in the workplace (6).

The third important topic of research on MSD are interventions to support work participation among workers with MSD, also known as tertiary prevention. The first paper in SJWEH that addressed this topic was published in 1991 (7), in which the effect of pre-employment medical examinations in a large occupational health service in The Netherlands was evaluated. The authors concluded that the several hundreds of thousands of pre-employment medical examinations performed each year in The Netherlands did not reduce absenteeism or work disability and therefore "it would appear to be tempting to end this practice" or "(as) an alternative approach (…) pay more attention to the possibility of providing information to applicants (eg, about health risks of the job and about possibilities for prevention)".

As the above papers show, much has been learned during the 50 years of research on (work-related) MSD published in SJWEH. Multiple efforts to prevent and manage MSD over the past five decades have been undertaken based on this knowledge. For instance, the European Union Directive 90/269/EEC addresses manual handling of loads, suggesting "minimum health and safety requirements for the manual handling of loads where there is a risk, particularly of back injury to workers". The EU-OSHA 2020-2022 *Healthy Workplaces – Lighten the Load* campaign tried to stimulate prevention and management of work-related MSD in all 27 European members states. In the United States, the National Institute for Occupational Safety and Health (NIOSH) has established Ergonomics Guidelines for Manual Handling, with guidance on how to reduce MSD in several high-risk industries including meatpacking, construction, and agriculture. Similarly, in Australia, the Work Health and Safety Act 2011 mandates that employers must actively manage the risks associated with work-related MSD.

• Despite these efforts, MSD continue to create significant personal and societal burdens worldwide and still constitute a major public health challenge, also given the aging working population. For example:The most common work-related health problems affecting European workers are MSD. In a continent-wide survey, roughly six out of every ten workers reported MSD in the last 12 months (8). There were large variations between European countries, with the lowest prevalence reported in Hungary (40%) and the highest in Finland (79%). The most common types of MSD reported by workers were back and upper limb pain;Work-related MSD put a large burden on individuals and society. According to the Global Burden of Diseases, Injuries, and Risk Factors Study, there were 126.1 million prevalent cases of work-related low-back pain in 2019, resulting in $216.1 billion in economic losses worldwide. Of this amount, $47.0 billion (22%) were healthcare costs and the remaining $169.1 billion (78%) were due to productivity losses (9);

MSD are the largest contributor to work productivity loss. A multi-cohort study from the United Kingdom, France, and Finland showed that the most common diagnoses for sickness absence were MSD (71 days per 10 person-years), followed by depressive disorders (27 days per 10 person-years), and external causes like injuries (13 days per 10 person-years) (10). In this paper, we provide an overview of 50 years of research on MSD published in SJWEH, organized around the three themes: (i) risk assessment, (ii) interventions to prevent work-related MSD, and (iii) interventions to support work participation among workers with MSD. By describing the evolution of research on MSD over the past half century, we aim to highlight the importance of continued research to understand, prevent, and manage work-related MSD and focus attention on topics for future research and actions towards prevention and improving work-participation.

## Methods

We performed a systematic literature search in PubMed to retrieve all papers on MSD published in SJWEH from the journal's launch in January 1975 to 22 January 2024.

### Search strategy and inclusion criteria

We used search terms for MSD, body regions, and risk factors to build a sensitive search strategy (supplementary material, URL, table S1). The retrieved papers were uploaded in the online screening tool Rayyan (Rayyan.ai). The first and last author independently assessed title and abstract to determine whether a paper fulfilled the primary inclusion criterion of addressing the topic of MSD. MSD were defined as conditions that affect the muscles, bones, joints, ligaments, tendons, and other supporting structures of the musculoskeletal system that may result in pain and loss of function. We also included conditions that do not necessarily have an origin in the musculoskeletal system, but have similar risk factors and symptoms (eg, pain and limitations in strength or movement). Such conditions include peripheral compression neuropathies and vascular disorders such as carpal tunnel syndrome and Raynaud’s syndrome. In case of disagreement or when no clear assessment could be made based on the abstract, the full paper was assessed for inclusion. Conflicts between the authors were discussed until consensus was reached.

### Data extraction

The second author labelled each paper according to its year of publication, country of origin, first author, study design, research topic, disease or complaint, corresponding body regions, and type of risk or prognostic factor(s) studied, using pre-defined categories. The number of citations was based on Web of Science (www.webofscience.com) and, if no data were available, on Scopus (www.scopus.com). The labelling was similar to the first paper in this series describing 50 years of research in SJWEH (11). Studies were also assigned to one of the three themes as described before: risk assessment, interventions to prevent work-related MSD, and interventions to support work participation among workers with MSD. Supplementary table S2 contains the data extraction scheme. To secure a sufficient reliable and valid labelling, the first, second and last author had a training session in which they independently assessed and then discussed five randomly selected papers. For the remaining papers, in case of doubt the second author discussed the assessment with the first and/or last author to achieve consensus.

### Data analysis

The labelling of all included papers was used to provide a systematic overview of what countries contributed to research in the journal and what specific MSD and themes were studied. Furthermore, an overview was provided of the body regions involved, the topics of the included studies, and risk factors assessed. Also, the most cited reviews and original research papers on MSD were presented, as were all interventions aimed at prevention of work-related MSD and support of work participation. After describing these data, the authors then discussed implications for future research.

## Results

**Figure 1:**
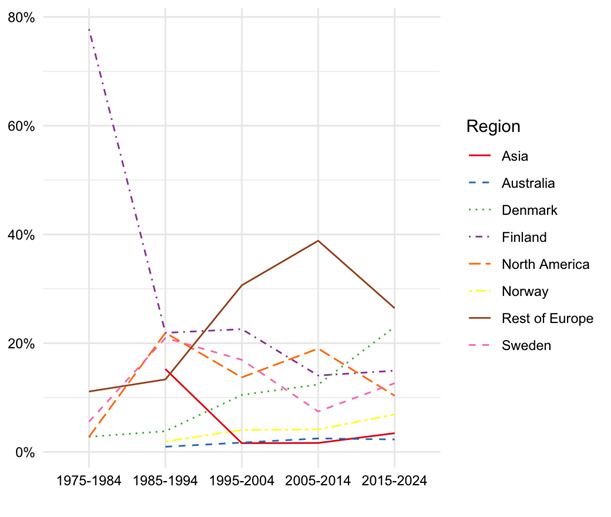
The percentage of papers on MSD published in the SJWEH per region over 10+year intervals plotted against the 5 decades of the journal. We depicted Scandinavian countries, rest of Europe, North America, Australia and Asia.

The search strategy resulted in 1056 papers, of which 474 were included after screening. These papers originated from 25 countries, mostly Finland (109 papers), followed by Sweden (65 papers), and The Netherlands (57). In the first decade of the Journal, nearly all papers were from Scandinavian countries (figure 1). Since then, authors from across the world have published papers in SJWEH, although the Scandinavian countries remained the most productive with 52% of all papers, followed by the rest of Europe (27%), North America (15%), Asia (5%), and Australia (1%). In the first decade, 38 papers were published on MSD, and then the number remained fairly constant with an average of 109 papers per decade (range 79–129).

### Types of MSD

**Figure 2:**
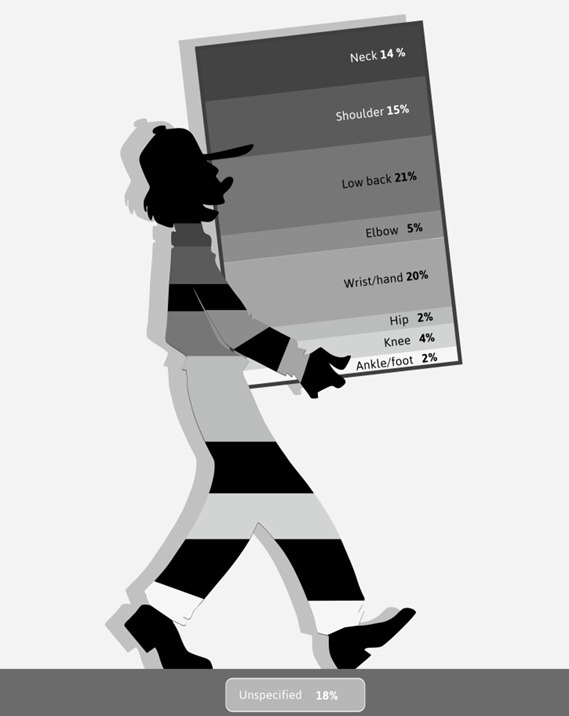
Body regions reported on in papers on MSD in the SJWEH over the 50 years, as a percentage of the total number of body regions reported on – with the possibility of more than one body region per paper and including a category ‘unspecified’ with 18%.

The low back (21%) and the wrist/hand (20%) were the most reported on body regions of MSD (figure 2). The lower extremities (hip, knee and ankle/foot) received the least attention with 8% of the papers published on this region. In the early years of SJWEH, most papers published were on Raynaud’s disease. The first paper on this topic was published in June 1975 (12). Up to and including 1990, more than half (57%) of the total number of papers on MSD addressed this disease. Therefore, Raynaud’s disease is the second most reported MSD on (11%) followed by carpal tunnel syndrome (6%). Four percent of the papers were on osteoarthritis. In total, 25 different diagnoses of MSD were reported and 39% of the studies made no distinction regarding a specific MSD. These studies reported on complaints due to MSD, while 8% of these papers used pain and 1% another symptom than pain like numbness or tingling.

### Topics

**Figure 3:**
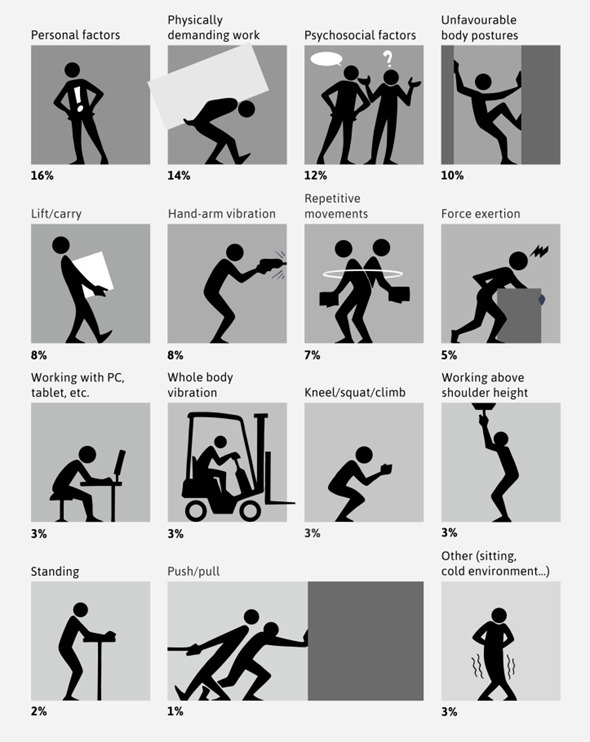
Risk factors reported on in papers on MSD in the SJWEH over the 50 years, as a percentage of the total number of risk factors reported on – with the possibility of more than one risk factor per paper.

The majority of papers in the SJWEH reported on risk factors (53%). The most commonly reported risk factors were personal risk factors for MSD like age, gender or smoking (16%, figure 3), followed by physically demanding work (14%) and psychosocial factors (12%). Other frequently reported physical risk factors were unfavourable body postures (10%), lifting and/or carrying (8%), hand-arm vibration (8%), repetitive movements (7%) and force exertion (5%).

Papers on interventions summed up to 14% of all papers published in SJWEH, 8% were on prevention of work-related MSD and 6% on work participation. All the other topics (supplementary table S2) like diagnostics of disease, exposure assessment of risk factors and studies on prognostic (personal and work-related) factors for work participation - were each reported on in 5–6% of the papers.

### Most cited papers and five observations

Considering table 1, describing the top ten most cited review and original research papers on MSD published in SJWEH, we made five observations:

1. Case definitions are key;
2. Over time, exposure criteria became more precise and increasingly driven by technology;
3. Unraveling the impact of work on low back pain;
4. Psychosocial risk factors matter; for some MSD probably more than for others; and
5. Conceptual models appear crucial in fostering multi-disciplinarity.

**Table 1:** The top 10 most cited review and original research papers on MSDs in the SJWEH over the past 50 years

Order and first author		Title	Year	Citations
**Reviews**				
1	Bongers et al (38)	Psychosocial factors at work and musculoskeletal disease	1993	854
2	Burdorf et al (42)	Positive and negative evidence of risk factors for back disorders	1997	513
3	Armstrong et al (43)	A conceptual model for work-related neck and upper-limb musculoskeletal disorders	1993	441
4	Hoogendoorn et al (44)	Physical load during work and leisure time as risk factors for back pain	1999	400
5	Sluiter et al (13)	Criteria document for evaluating the work-relatedness of upper-extremity musculoskeletal disorders	2001	370
6	Ariëns et al (45)	Physical risk factors for neck pain	2000	357
7	van Rijn et al (19)	Associations between work-related factors and specific disorders of the shoulder–a systematic review of the literature	2010	279
8	MacEachen et al (46)	Systematic review of the qualitative literature on return to work after injury	2006	254
9	Riihimäki (33)	Low-back pain, its origin and risk indicators	1991	209
10	Lötters et al (30)	Model for the work-relatedness of low-back pain	2003	184
**Original research**				
1	Punnett (47)	Back disorders and nonneutral trunk postures of automobile assembly workers	1991	391
2	Bernard et al (48)	Job task and psychosocial risk factors for work-related musculoskeletal disorders among newspaper employees	1994	314
3	Veiersted et al (49)	Electromyographic evaluation of muscular work pattern as a predictor of trapezius myalgia	1993	262
4	Wiktorin et al (50)	Validity of self-reported exposures to work postures and manual materials handling. Stockholm MUSIC I Study Group	1993	227
5	Viikari-Juntura et al (51)	Validity of self-reported physical work load in epidemiologic studies on musculoskeletal disorders	1996	193
6	Chiang et al (52)	Prevalence of shoulder and upper-limb disorders among workers in the fish-processing industry	1993	193
7	Dale et al (53)	Prevalence and incidence of carpal tunnel syndrome in US working populations: pooled analysis of six prospective studies	2013	190
8	Houtman et al (54)	Psychosocial stressors at work and musculoskeletal problems	1994	176
9	Blangsted et al (55)	One-year randomized controlled trial with different physical-activity programs to reduce musculoskeletal symptoms in the neck and shoulders among office workers	2008	171
10	Knave et al (56)	Work with video display terminals among office employees. I. Subjective symptoms and discomfort	1986	170

*Case definitions are key.* Already in the first papers on MSD in SJWEH, authors plead to be as specific as possible regarding the case definition of the specific MSD or symptom assessed. Case definitions may differ in precision, whether they were self-reported or clinically assessed, and depending on their purpose. For instance, definitions varied when the assessment was to determine treatment options for individual workers, financial compensation, or assessing risk factors or the effect of preventive measures in a large study sample.

A landmark paper on case definitions is the criteria document for evaluating the work-relatedness of upper-extremity MSD (13). The paper was the result of a European joint program for working life research in Europe undertaken by the three Swedish confederations of employees and the Swedish National Institute for Working Life. The methods used involved a project team of European experts, review of the literature, survey of members of an organization for these disorders, and finally a workshop among experts from 14 European countries.

Until then, a variety of umbrella terms was used to describe upper-extremity MSD, which were thought to be related to repeated micro-trauma, often involving prolonged computer work. These terms included repetitive strain injury, occupational overuse syndrome, occupational cervicobrachial disorder, and cumulative trauma disorder. They all assumed a link between the clinical disorder(s) and the suspected causal factor or mechanism of injury. The criteria document was developed to overcome "… the considerable uncertainty and even controversy about the extent and etiology of these problems, the contribution of work and non-work risk factors to their development and resolution, the criteria used to diagnose them, the outcomes of various treatment methods, and the appropriate strategies for intervention and prevention". The criteria document sets case definitions for 11 specific and one non-specific disorder of the upper extremities, which were defined according to a duration of the complaints and signs and symptoms. In addition, specific work-related criteria were defined, involving factors like postures, movements, hand force applied, use of vibrating tools and/or working in a cold environment. Also, non-physical factors like work-rest schedules and psychosocial demands were defined. As a follow-up, a systematic literature review and an international Delphi study were performed to arrive at case definitions for similar and other prevalent work-related MSD, namely work-related low-back pain, lumbosacral radicular syndrome, subacromial pain syndrome, carpal tunnel syndrome, lateral and medial elbow tendinopathy, and knee and hip osteoarthritis (14, 15). Importantly, these case definitions allowed linkage of commonly recognized clinical MSD to newly identified work-related risk factors (16–18).

*Over time, exposure criteria became more precise and increasingly driven by technology.* Knowledge about the extent to which a risk factor contributes to the onset or worsening of a specific MSD has improved greatly in the last 50 years. In the first SJWEH papers on MSD, exposure assessment was often done in crude categories, eg, in terms of professions like drillers or sandblasters versus non-exposed reference groups, or in terms of performing an activity like using pneumatic hammers without details about the exact exposure (12). In later papers, the exposure criteria became more distinct. An example is the often cited paper by van Rijn and colleagues (19) in which the occurrence of subacromial impingement syndrome was shown to be associated with "force requirements >10% maximal voluntary contraction, lifting >20 kg >10 times/day, and a high-level of hand force >1 hour/day". The certainty on the work-relatedness of these risk factors for sub-acromial impingement syndrome was further increased by a recent paper by Dalbøge and colleagues (20). Based on a register-based cohort study on surgery for subacromial impingement syndrome among the entire Danish working population and using a job exposure matrix, exposure–response relationships were found for single and combinations of risk factors like arm-elevation, force, and the cumulative exposure of one or two other mechanical exposures.

In the upcoming years, we expect that the use of motion sensors will further increase both the precision of measurements and the strength of the evidence linking these and other work-related risk factors to MSD (21, 22). The sensors can measure exposure over time and thus enable a more detailed exposure characterization for both short- and long-term health effects (23–25). Especially if international collaborations create common exposure metrics and analytical strategies for epidemiological studies, preferably related to evidence-based risk factors for work-related MSD, progress can be expected (26). Also the individual worker might benefit from these sensors given the advantages for workplace risk assessments, worker health surveillance, occupational disease assessments, and evaluating the effectiveness of a preventive measure. For instance, when sensors incorporate algorithms as used in health impact assessment, workers can get real-time feedback on whether their work puts them at risk for specific MSD like low-back pain (27–29) or knee osteoarthritis (28).

*Unraveling the impact of work on low-back pain.* As can be seen in table 1, four of the ten most cited reviews are on work-related risk factors for low-back pain, while the most cited original research paper is on the same topic. All these papers provide evidence for an association between low-back pain and work-related risk factor(s), including manually lifting and carrying loads, bending and twisting of the trunk and whole-body vibration. To support practitioners in evaluating the relative contribution of these work-related risk factors to the occurrence of low-back pain in an individual worker, Lötters and colleagues (30) developed a practical tool that translates the population-based attributable fraction of a single or combination of work-related risk factors into an individual attributable risk. Recently, a systematic review and meta-analyses confirmed that "moderate evidence of an association was found for lifting and carrying loads, non-neutral postures, and combined mechanical exposures" with (chronic) low-back pain with statistically significant odds ratios of 1.5–2.2 (31). Unravelling the impact of work on low-back pain remains important given that it is the leading cause of disability worldwide (32). Additional reasons for the continuous effort are the prevailing lack of clear understanding of the dynamic nature (33, 34), the multi-factorial etiology of low-back pain and an apparent relatively low population attributable fraction for work-related factors compared to other MSD (30, 35). In addition, misclassification of exposure attenuates the estimate of the work-related risk. Recent studies try to overcome misclassification of exposure by carefully selecting their research design and, for instance, by specifying acute (36) versus chronic low back pain (37).

*Psychosocial risk factors matter; for some MSD probably more than for others.* Psychosocial risk factors are often studied to better understand the etiology of work-related MSD (figure 3, table 1). The most cited SJWEH paper by Bongers and colleagues (38) presented a model describing how psychosocial factors, like job demands and control and social support, might influence physical work demands (‘mechanical exposure’) and physical and behavioral health indicators, and thereby influence MSD and their chronicity, sick leave and work disability. The authors provided an overview of the existing knowledge on these mechanisms for low-back pain, neck and shoulder pain, and MSD in general and concluded that the evidence was inconclusive. More recent research has shown that for clinically assessed specific MSD, psychosocial risk factors might play a differential role depending on the specific MSD. For instance, on the one hand, a review by van der Molen and colleagues (35) concluded that there was no association of social support, decision latitude, job control and job security with specific shoulder disorders. Similarly, a review by Jahn and colleagues (39) showed no significant associations between several psychosocial risk factors, like job stress, support and satisfaction, and chronic low-back pain. On the other hand, original studies by Harris-Adamson and colleagues (40) concluded that job strain and forceful hand exertion were both independent risk factors for carpal tunnel syndrome and Zhou and colleagues (41) concluded that shift work can be seen as an independent risk factor for knee osteoarthritis. Future research should assess how psychosocial risk factors influence workplace physical exposures and the importance of psychosocial factors at different stages of disease and type of disease. The relative importance of physical and psychosocial risk factors are likely different for the outcome of initial pain symptoms than for the outcome of chronic work disability.

*Conceptual models appear crucial in fostering multi-disciplinarity.* The fact that more than half of the papers on MSD in SJWEH over the past 50 years are on the topic of risk assessment is also reflected by highly cited papers on corresponding conceptual models (38, 43) and the already mentioned research framework (2). These papers facilitate research from different disciplines and with different methodologies and contribute to a better understanding of disease and risk factors, which may ultimately increase both our understanding and the effectiveness of prevention (2). As far as we are aware, no such specific stepwise conceptual model or framework exists regarding our third aim – interventions to support work participation among workers with MSD. The World Health Organization's framework for the International Classification of Functioning, Disability and Health (ICF) might be a good starting point as could be the models promoted by Loisel, Evanoff and their respective colleagues (57, 58). To foster multi-disciplinarity, we preferably should make this model or framework more work(er) and disease specific. A first step might be that intervention studies on work participation should clearly define their conceptual model and describe how relevant prognostic factors for work participation are taken into account (59, 60).

### Interventions to prevent work-related MSD and support work participation among workers with MSD

Supplementary table S3 shows the design, outcome measures and conclusion of the intervention studies both to prevent work-related MSD and support work participation of workers with MSD. We included 40 papers evaluating interventions: 21 on preventing work-related MSD and 19 on supporting work participation among those with MSD. Most studies (N=26) adopted the randomized controlled trial (RCT) design. The content of the interventions varied widely: 17 interventions were directed at the worker-level (micro-level) [eg, in the early years of the journal pharmaceutical treatment to diminish the effect of a work-related exposure (61) or individual fitting of shoes among newspaper carriers (62)]; 22 interventions were directed at the workplace (meso-level) and focused on both work-related and personal factors including workplace improvements, physical exercise and reducing working hours (63) or training health care professionals in guideline-oriented biopsychosocial management of low back pain (64). One intervention (65) was focused at the macro-level and evaluated the Danish national *Job & Body* campaign. More than 70% of the papers evaluating interventions reported in their conclusion that the intervention showed a positive effect, although these positive results might be biased due to issues like publication bias or reporting on non-primary outcomes.

When considering intervention studies for prevention of work-related MSD, the minority of studies addressed a specific MSD; three focused on low-back pain (66–68) and four on Raynaud’s syndrome (61, 69–71). Out of the studies on low-back pain, two reported no effects: one focused on a participatory ergonomics program called *Stay@Work* (66) and the other on a brief workplace cognitive and exercise intervention (67). Both studies explained their lack of statistically significant findings given that the intervention had only a small effect on reducing the exposure to the work-related risk factors for low-back pain. The significant effect of the third low-back pain intervention study among high-risk office workers was primarily due to a substantial reduction in exposure to the work-related risk factors for low back pain (68). The intervention consisted of active breaks and postural shifts facilitated by a custom-designed inflatable seat pad, including a smartphone application. Both the number of active breaks and postural shifts were larger than previously reported in similar studies on low-back pain. Sufficient exposure reduction for established work-related risk factors regarding Raynaud’s disease also explained why strict regulations regarding the maximum cycle time (ten minutes), the number of hours per day (four), days a week (four) and days per year (120) using better designed chainsaws and an age restrictions (55 years) "… can completely prevent the vibration syndrome even if the total operating time is appreciably lengthened" (71).

When looking at intervention studies to support work participation among workers with MSD, an early multi-faceted approach addressing both personal and work-related factors as part of the care-as-usual for medically verified MSD seems effective. An example is an intervention consisting of a physician contacting the worker’s supervisor and an occupational physiotherapist conducting an ergonomic assessment at the worksite for workers with clinically assessed upper-extremity disorders (72, 73). In this intervention, the suggestions for improvements by the occupational physiotherapist were discussed together with the employee and the supervisor, the latter then made the final decision on the technical and administrative changes at work. The authors of these papers concluded that "… an early ergonomic intervention in addition to adequate medical care help to reduce work-related productivity loss associated with upper-extremity disorders compared to medical care on its own" (72) and "…an early ergonomic intervention reduces sickness absence due to upper-extremity or other musculoskeletal disorders" (73). The intervention seemed especially promising among workers with no keying at work in physically demanding jobs. Moreover, given the positive effects of workplace strength training on work ability (74) and pain (75), it would be relevant to know whether such interventions would have further improved productivity outcomes and reduced sickness absence.

## Discussion

Based on the past 50 years of research on MSD in the Journal, we feel that we have progressed in the themes of better case definitions and more precise work-related exposure criteria, especially for physically demanding work. In addition, the journal has provided evidence on interventions for both prevention of work-related MSD and support of work participation of workers with MSD, all based on good quality research. Based on the above, we hope and are confident that in the upcoming years more research on MSD will focus on interventions, whole-system approaches and musculoskeletal health.

### More intervention studies are needed

The last MSD intervention study published in SJWEH dates from 2021. Despite the gain in more knowledge about work-related exposures for prevention of MSD and prognostic factors for work participation among workers with MSD, more intervention studies are needed. We encourage authors to incorporate a theory-driven approach with testable hypotheses for the effective components of their interventions. By doing so, we could ultimately gain a clearer understanding of the transition of asymptomatic workers to those experiencing MSD symptoms, whether or not they (i) seek treatment, (ii) experience work disability, and (iii) can participate fully in their work (58). Perhaps the variety in healthcare and workplace interventions and the number of work-related and non-work-related risk and prognostic factors as well as workplace policies and regulations is not as extensive as expected to maintain a healthy worker and workforce. When incorporating specific MSD and utilizing internationally accepted case definitions, these findings can be readily integrated into treatment plans and guidelines by health professionals and clinicians across various disciplines (76). Therefore, we should not only rely on an RCT design but also stimulate the use of alternative designs such as interrupted time-series (77), which allows the use of routinely collected MSD and participation data from health registries, company medical records, workers health surveillance, or cohorts like the ones from the Network on the Coordination and Harmonisation of European Occupational Cohorts (OMEGA-NET).

### Adopt whole-system approaches to improve prevention of work-related MSD

Work-related MSD still constitute a major public health challenge, despite the availability of potential interventions. Guidelines and regulations implemented in several countries seem not to have resulted in substantial societal impact. It is not surprising that MSD persist when their associated risk factors, like physically demanding work, remain highly prevalent. A systematic review performed for the World Health Organisation and International Labour Organisation in which the work-related burden of diseases and injuries was estimated showed that, for instance, the pooled prevalence of physically demanding work in the European region is 76% with little variation across countries (95% confidence intervals 69–84%) (78). In line with the research framework for the development, evaluation, and implementation of interventions preventing work-related musculoskeletal disorders (2), an extra step might be warranted. This proposed 7^th^ step should provide more insight in the actual drivers that truly improve the prevention of work-related MSD, with a particular focus on the meso (workplace) and macro (society) levels. Therefore, performing a so-called whole-system approach becomes imperative to better understand these drivers (79, 80), which probably involve coordinated actions across a broad range of disciplines and stakeholders, different levels of public and private governance, and throughout the life course of workers.

### Focus on musculoskeletal health instead of only disease

Existing literature on work and health has primarily focused on elucidating the adverse outcomes associated with work, including the presence of MSD and other diseases, disorders, or associated symptoms like pain. Surprisingly, no studies addressed the fundamental concept of musculoskeletal health itself. Understanding the factors that contribute to musculoskeletal health is probably advantageous for a better understanding of preventing the negative consequences of MSD (81, 82). Here, consensus among researchers, practitioners, and workers regarding the construct of musculoskeletal health is crucial. A good example is the Arthritis Research UK Musculoskeletal Health Questionnaire (83), which encompasses domains beyond mere physical functioning and pain, including work and social interference, physical activity, independence, and confidence in self-management. This outcome measure ensures that interventions may benefit all workers, with or without an MSD. This not only presents advantages for conducting intervention studies, as a larger pool of workers may find participation beneficial, but also holds promise for research. The utilization of the concept of musculoskeletal health as an outcome measure could also prove invaluable in studies aimed at both preventing work-related MSD and promoting work participation across diverse populations and workplace settings around the world.

## Supplementary material

Supplementary material
