# Supplementary material for: What have we learned about risk assessment and interventions to prevent work-related musculoskeletal disorders and support work participation?
Source: Scand J Work Environ Health. 2024 Jun 27;50(5):317–28. doi: 10.5271/sjweh.4172 (PMC11214778; doi:10.5271/sjweh.4172)
Supplement: Supplementary material [file SJWEH-50-317-S001.pdf]

What have we learned about risk assessment and interventions to prevent work-related musculoskeletal disorders and support work participation?1

P. Paul F.M. Kuijer, PhD,[2] Sten van der Wilk, BSc, Bradley Evanoff, MD, MPH, PhD, Eira Viikari-Juntura, MD, PhD, Pieter Coenen, PhD

1. Supplementary material
2. Correspondence to P. Paul F.M. Kuijer, Amsterdam UMC, Department of Public and Occupational Health, Amsterdam, The Netherlands [Email: p.p.kuijer@amsterdamumc.nl]

Table S1 The search strategy used in PubMed to retrieve studies on musculoskeletal disorders in the Scandinavian Journal of Work, Environment and Health until January 22nd 2024.

	**Search terms**	**Results**
1	Scandinavian journal of work, environment health [Journal]	3694
2	musculoskeletal diseases [MeSH terms]	1.225.787
3	ache, low back [MeSH Terms] OR elbow [MeSH Terms] OR ache, neck [MeSH Terms] OR knee [Title/Abstract] OR shoulder [Title/Abstract] OR hip [Title/Abstract] OR ankle [Title/Abstract] OR wrist [Title/Abstract] OR hand [Title/Abstract] OR foot [Title/Abstract]	1.059.742
4	repeti*[Title/Abstract] OR Posture [Title/Abstract] OR Pull [Title/Abstract] OR Push* [Title/Abstract] OR Vibration*[Title/Abstract] OR Lift* [Title/Abstract] OR physical [Title/Abstract] OR demands [Title/Abstract] OR workload [MeSH terms]	1.350.622
5	biomech* [Title/Abstract]	89.871
6	2 OR 3 OR 4 OR 5	3.322.578
7	1 AND 6	1.057

Table S2 Scheme used for the labelling of the papers on musculoskeletal disorders

Year of publication	Country, 1st author	Study design	Topic (more options can apply)	Disease (more options can apply)	Body region (more options can apply)	Risk factors (more options can apply) if topic 3-7	Prognostic factor – if topic 9	Number of citations
		Cross-sectional	1.Disease - Diagnostics	Raynaud	Neck	Physically demanding work, unspecified	Physically demanding work, unspecified	
		Prospective cohort	2.Prevalence/Incidence	Low back pain	Shoulder	Whole Body Vibration	Psychosocial factors	
		Retrospective cohort	3.Etiology - Risk factors	Lumbosacral radiculopathy	Elbow	Hand-arm Vibration	Other…	
		Case-control	4.Etiology – Mechanisms / pathofysiology	Subacromial Pain Syndrom	Wrist/hand	Lift/carry		
		RCT	5.Risk factors - Exposure assessment	Carpal Tunnel Syndrome	Low back	Push/pull		
		Experimental / Laboratory study	6.Prevention – Interventions exposure	Rheumatism	Hip	Working above shoulder height		
		Review	7.Prevention – Interventions disease	Elbow tendinopathy	Knee	Kneel/squat/climb		
		Editorial / Letter to Editor	8.Work participation – personal prognostic factors	Osteoarthritis CMC-1 / thumb base	Ankle/foot	Standing		

		…	9.Work participation – work-related prognostic factors	Osteoarthritis knee		Repetitive movements		
			10.Work participation – Interventions workplace	Osteoarthritis hip		Unfavourable body postures		
			11.Work participation – Interventions other	Pain		Working with pc, tablet, etc		
				Other symptoms		Force exertion		
				…		Psychosocial factors		
						Other		

Table S3 Overview of the original research papers (sorted by year of publication) on intervention studies to prevent work-related musculoskeletal disorders and to support work participation

First author	Year	Design	Intervention	Primary outcome	Secondary outcome	Conclusion
Pyykkö [1]	1982	Prospective cohort	The effect of clonidine, a centrally acting drug diminishing the sympathetic activity of peripheral nerves, was examined among forest workers with vibration-induced white fingers	Frequency of attacks and recovery time	Subjective evaluation of overall effect of the therapy	No significant differences were discerned between the active and placebo groups in respect to either recovery time after an attack or frequency of attacks. Neither did the subjective evaluation of the overall effectiveness of the

						drug show any differences between the groups
Nasu [2]	1986	Retrospective cohort	Effects of batroxobin (a defibrinogating drug) on the peripheral circulatory disturbance of patients with vibration syndrome	Blood flow, skin temperature, finger plethysmography and nail compression	Subjective symptoms	The results suggest that defibrinogation by batroxobin is useful for improving the peripheral circulatory disturbance of the vibration syndrome
Nasu [3]	1986	Retrospective cohort	Most of the patients received treatment combining physical and drug therapy	Subjectively and objectively measured symptoms of vibration syndrome		There is a limitation (in terms of period in which effectiveness can be observed) in the treatment of disturbances related to the vibration syndrome
Saito [4]	1987	Retrospective cohort	Work conditions for chain-saw workers	Symptoms	Skin temperatures, vibratory sense thresholds, recovery time of nail blood colour in the nail, and the pain sense	Adequate restrictions on the operating time of the chain saw and on the age of workers can completely prevent the vibration syndrome even if the total operating time is appreciably lengthened
Brisson [5]	1999	RCT	An ergonomic training program on workstation changes and on the prevalence of	Three specific postural stressors: (i) twisted neck, (ii) inappropriate height of visual target,	Musculoskeletal disorders	Improvements in postural stressors occurred more frequently in the experimental group, and

			musculoskeletal disorders among video display unit users at a large university	and (iii) broken (bent) hand-wrist line		improvements in musculoskeletal disorders occurred in the experimental group (among the workers under 40 years)
Ketola [6]	2002	RCT	Intensive ergonomic approach and education on workstation changes	Workstation changes and musculoskeletal disorders	Strain levels, prevalence of pain	Both the intensive ergonomics approach and education in ergonomics help reduce discomfort in VDU work
Torkki [7]	2002	Case-control	Individually fitted sports shoes against overuse injuries to the lower limb among newspaper carriers	Lower-limb pain intensity, duration of pain, ability to work, foot fatigue, costs of foot care, satisfaction with work shoes, fall accidents at work and sick leave related to lower-limb disorders.		Individually adjusted shock-absorbing shoes offer slight health benefits for lower-limb overuse injuries.
Smedley [8]	2003	Prospective cohort	Implementation of an ergonomic intervention on the revision of the hospital-wide manual-handling policy to minimize unassisted patient handling and	Implementation of the intervention	Musculoskeletal symptoms	There was some reduction in the frequency with which patient-handling activities were carried out without the use of mechanical aids. However, the changes were modest

			exposure to high-risk nursing tasks			in scale. There was no reduction in the prevalence of symptoms at either hospital
van den Heuvel [9]	2004	RCT	The use of a software program stimulating workers to take regular breaks	Recovery of work-related neck and upper-limb disorders	Sick leave	The use of a software program stimulating workers to take regular breaks contributes to perceived recovery from neck or upper-limb complaints
Mattila [10]	2004	RCT	Lifestyle intervention to control hypertension and its effect on neck, shoulder, elbow, and wrist symptom	Neck, shoulder, elbow, and wrist symptoms and disability	Weight, body mass index, waist and hip circumferences, perceived depressive mood, and physical activity	Lifestyle intervention to control hypertension has a favourable impact on perceived disability due to neck pain
Luijsterburg [11]	2005	Prospective cohort	The introduction of devices for raised bricklaying	Physical work load	Musculoskeletal symptoms and sickness absence	This study shows that the introduction of an ergonomic improvement in the construction industry may reduce physical load and the incidence of sickness absence

Blangsted [12]	2008	RCT	Three intervention groups: one with specific resistance training (SRT) of the neck-shoulder region (N=180), one with all-round physical exercise (APE) (N=187), and one which acted as a reference group, which was informed about general health-promoting activities but did not include a physical activity program (N=182)	Neck-shoulder symptoms	Work ability index (WAI) and sick leave	Different physical-activity interventions were successful in reducing neck-shoulder symptoms, and SRT was superior to APE in the primary prevention of such symptoms
Von Thiele Schwarz [13]	2008	RCT	Worksite interventions, physical exercise and reduced workhours	Physical activity and exercise	Glucose, high-density lipoprotein, waist-to-hip ratio, work ability, general symptoms and upper-extremity disorders	The two interventions had small and varied effects on biomarkers and self-reports of different aspects of health among women
Welch [14]	2009	Prospective cohort	Receiving some type of job accommodation	Leaving work by the time of the 1-year follow-up	-	Job accommodation was associated with a reduced likelihood of subsequently leaving roofing for health-related reasons

Martimo [15]	2010	RCT	The intervention consisted of a physician contacting the worker's supervisor and an occupational physiotherapist conducting an ergonomic assessment at the worksite.	Self-assessed upper-extremity disorders-related productivity loss	-	Early ergonomic intervention, in addition to adequate medical care, is effective in preventing and restoring self-reported productivity loss associated with UED
Driessen [16]	2011	RCT	Stay@Work participatory ergonomics program to prevent low-back and neck pain.	Neck and low-back pain	Pain intensity and pain duration	PE was not effective on any of the outcomes
Jay [17]	2011	RCT	Kettlebell training to improve musculoskeletal and cardiovascular health	Musculoskeletal pain	Muscle strength, aerobic fitness	Worksite intervention using kettlebell training reduces pain in the neck/shoulders and low back and improves muscle strength of the low back among adults from occupations with a high prevalence of reported musculoskeletal pain symptoms
Shiri [18]	2011	RCT	Workplace ergonomic improvements	Upper-extremity symptoms	Sickness absences due to upper-extremity or other	Early ergonomic intervention reduces sickness absence due to

					musculoskeletal disorders	upper-extremity or other musculoskeletal disorders.
Viikari-Juntura [19]	2012	RCT	Part- time (i.e. workload was reduced by restricting work time by about a half) or full-time sick leave. Remaining work tasks were modified when necessary, as specified in a “fit note” from the physician	Time to return to regular work activities and sickness absence during 12-month follow-up	-	Early part-time sick leave may provide a faster and more sustainable return to regular duties than full-time sick leave among patients with musculoskeletal disorders.
Oude Hengel [20]	2013	RCT	The intervention consisted of two individual training sessions with a physical therapist aimed at lowering the physical workload, a rest-break tool to improve the balance between work and recovery, and two empowerment training sessions to increase the worker's influence at the worksite.	Work ability	Physical and mental health status, and musculoskeletal symptoms, sick leave	The prevention program seemed to result in a beneficial but not statistically significant decline in the prevalence of musculoskeletal symptoms and long-term sick leave among construction workers, but showed no effects with regard to work ability, physical health, and mental health.
Schoenfisch [21]	2013	Retrospective cohort	Implementation of "minimal manual lift" policy and mechanical lift equipment	Musculoskeletal symptoms and worker’s compensation	-	Institutional-level changes at the time of the intervention likely influenced observed results with findings only partially

						consistent with an intervention effect
Shiri [22]	2013	RCT	Part- (halving working time) or full-time sick leave groups	Pain intensity and interference with work and sleep, region-specific disability due to MSD, self-rated general health, health-related quality of life, productivity loss, depression, and sleep disturbance	-	Part-time sick leave did not exacerbate pain-related symptoms and functional disability, but improved self-rated general health and health-related quality of life in the early stage of work disability due to MSD
Vermeulen [23]	2013	RCT	The new RTW program was aimed at making a consensus-based RTW action plan with the possibility of a temporary (therapeutic) workplace	Sustainable RTW	Quality-adjusted life years (QALY) and Healthcare utilization	The cost-effectiveness analyses showed that the new intervention was more effective but also more costly than usual care (ie, to gain RTW one day earlier in the participatory RTW program group approximately 80 euros needed to be invested).
Cantley [24]	2014	Prospective cohort	Ergonomic hazard control initiative, undertaken as part of a company ergonomics standard	MSD	-	The intervention was associated with MSD and injury risk reduction among workers in jobs with HC implemented.

Rantonen [25]	2014	RCT	The intervention group received the Back Book, an information booklet on how to manage low back pain, with an additional face-to-face review of the booklet by an OH nurse. The control group received the booklet only.	Physical impairment (Roland-Morris 18-item (RM-18) Disability Questionnaire), low back pain (VAS 100 mm), health-related quality of life [15-dimensional quality of life (15-D)] during two years and sickness absence	-	Face-to-face patient information by an OH nurse in addition to a booklet was not more effective than the booklet alone in treating employees with mild low back pain in an OH setting.
Sundstrup [26]	2014	RCT	10 weeks of either strength training for the shoulder, arm, and hand muscles (3 times per week, 10 minutes per session) or ergonomic training (usual care control group)	Work ability index (WAI)	-	Implementation of strength training at the workplace prevents deterioration of work ability among manual workers with chronic pain and disability exposed to forceful and repetitive job tasks
Jakobsen [27]	2015	RCT	Workplace physical exercise on musculoskeletal pain among healthcare workers	Pain intensity	Muscle strength and use of analgesics	Workplace physical exercise is more effective than home-based exercise in reducing musculoskeletal pain
Chaléat-Valayer [28]	2016	RCT	A light exercise program, initiated in the workplace and continued at home. The intervention comprised three	Recurrence of low-back pain	Psychological, physical and functional outcomes.	The results could not confirm that a brief workplace-based cognitive and exercise intervention,

			steps: (i) a 2-hour education session, (ii) five weekly 90-minutes exercise training sessions in the workplace, and (iii) a home-based self-managed exercise program		Healthcare utilization	followed by a home-based exercise program, is effective in secondary prevention to reduce low back pain recurrence with sick-leave among healthcare workers
Danquah [29]	2017	RCT	Take a Stand! is an intervention aimed at reducing sitting at work. The Take a Stand! intervention included five elements: (i) appointment of local ambassadors and management support, (ii) environmental changes, (iii) a lecture, (iv) a workshop aiming at ensuring local adaptation at individual, office and workplace level, and (v) e-mails and text messages.	Musculoskeletal pain	-	Take a Stand! reduced neck-shoulder pain after three months and total pain score after one and three months among office workers, but not neck-shoulder pain after one month or pain in the back and extremities
Viikari-Juntura [30]	2017	Quasi-experimental study	The use of part-time sick leave at the early (first 12 weeks) stage of work disability	Sustained return to work (RTW) and overall work participation	-	The use of part-time sick leave during the first three months of SA enhances RTW and overall work participation during two

						years among persons with mental disorders and musculoskeletal diseases.
Andersen [31]	2018	Prospective cohort	A national campaign in Denmark targeted public sector employees with a mixture of networking activities, workplace visits, and a mass media outreach with topics related to job and body (e.g., musculoskeletal pain, movement and work) and creating balance between demands at work and physical capacity	Beliefs about musculoskeletal pain	-	During the national campaign, beliefs about musculoskeletal pain and work were more positive
Jakobsen [32]	2019	RCT	Participatory organizational intervention for improved use of assistive devices in patient transfer.	Use of assistive devices	Musculoskeletal pain	The intervention did not result in more frequent use of the necessary assistive devices but led to more general use of assistive devices, as well as increased communication and guidance

Leinonen [33]	2019	Prospective cohort registry based study	Vocational rehabilitation	Proportion of time spent at work	-	Vocational rehabilitation after musculoskeletal- or mental-related work disability showed modest effectiveness on work participation
Pereira [34]	2019	RCT	An individualized workstation ergonomics intervention, combined with 12 weeks of either workplace neck-specific exercises or health promotion information.	Health-related productivity loss	Sickness presenteeism/ absenteeism	A workplace intervention combining ergonomics and neck-specific exercise offers possible benefits for sickness presenteeism and health-related productivity loss among a general population of office workers and sickness absenteeism for office workers with neck pain in the longer-term.
Viikari-Juntura [35]	2019	Quasi-experimental study	Partial sickness beneficiaries and a matched group of full sickness beneficiaries	Social security costs	-	Part-time instead of full-time sick leave, at the early stage of work disability due to musculoskeletal diseases or mental disorders, leads to considerable social security cost savings during two years, in correspondence with

						increased work participation
Gismervik [36]	2020	RCT	Inpatient multimodal occupational rehabilitation (I-MORE) I-MORE included ACT, physical exercise, work-related problem solving and creating a return to work plan	Sickness absence	-	Among individuals on long-term SA due to musculoskeletal and common mental health disorders, a 3.5-week I-MORE program reduced SA compared with 6 weekly sessions of O-ACT in the year after inclusion
Rasmussen [37]	2020	RCT	20-week workplace participatory ergonomic intervention among childcare workers on physical exertion and musculoskeletal pain	Self-rated physical exertion and musculoskeletal pain	self-reported sickness absence due to musculoskeletal pain, self-efficacy, need for recovery, and employee involvement	This 20-week training for a participatory ergonomic intervention in childcare workers did not show effects on physical exertion and musculoskeletal pain, but was both feasible and effective in reducing musculoskeletal pain-related sickness absence
Skagseth [38]	2020	RCT	A workplace intervention (WI) added to an inpatient multimodal occupational rehabilitation program (I-MORE)	Sickness absence	-	No evidence in favour of I-MORE+WI compared to only I-MORE for long-term sickness absent individuals with musculoskeletal-,

						common mental- or unspecified disorders.
Ryynänen [39]	2021	RCT	Brief training in the guideline-oriented biopsychosocial management of low-back pain in occupational health services	Disability (Oswestry Disability Index, ODI)	-	Brief training in guideline-oriented biopsychosocial management of low back pain for occupational health professionals did not appear to be effective in reducing patients' symptom over one-year follow-up compared to treatment as usual
Waongenngarm [40]	2021	RCT	Promotion of active breaks and postural shifts preventing new onset of neck and low-back pain	Neck and low-back pain	Neck and low-back pain disability	Interventions to increase either active breaks or postural shifts reduced new onset of neck and low-back pain among high-risk office workers

References

1. Pyykkö I, Färkkilä M. Clonidine in the treatment of vibration-induced white fingers. Scand J Work Environ Health. 1982; 8(4):294-299.
2. Nasu Y. Defibrinogating therapy for peripheral circulatory disturbance in patients with vibration syndrome. Scand J Work Environ Health. 1986; 12(S4):272-276.
3. Nasu Y, Ishida K. Follow-up study of patients with vibration syndrome in Japan. Scand J Work Environ Health. 1986; 12(S4):313-319.
4. Saito K. Prevention of the hand-arm vibration syndrome. Scand J Work Environ Health. 1987; 13(4):301-304.
5. Brisson C, Vinet A, Vezina M, Gingras S. Effect of duration of employment in piecework on severe disability among female garment workers. Scand J Work Environ Health. 1989; 15(5):329-334.

6. Ketola R, Toivonen R, Häkkänen M, Luukkonen R, Takala EP, Viikari-Juntura E, et al. Effects of ergonomic intervention in work with video display units. Scand J Work Environ Health. 2002; 28(1):18-24.
7. Torkki M, Malmivaara A, Reivonen N, Seitsalo S, Laippalo P, Hoikka V. Individually fitted sports shoes for overuse injuries among newspaper carriers. Scand J Work Environ Health. 2002; 28(3):176-183.
8. Smedley J, Trevelyan F, Inskip H, Buckle P, Cooper C, Coggon D. Impact of ergonomic intervention on back pain among nurses. Scand J Work Environ Health. 2003; 29(2):117-123.
9. van den Heuvel SG, Ariëns GA, Boshuizen HC, Hoogendoorn WE, Bongers PM. Prognostic factors related to recurrent low-back pain and sickness absence. Scand J Work Environ Health. 2004; 30(6):459-467.
10. Mattila R, Malmivaara A, Kastarinen M, Kivelä SL, Nissinen A. Effects of lifestyle intervention on neck, shoulder, elbow and wrist symptoms. Scand J Work Environ Health. 2004; 30(3):191-198.
11. Luijsterburg PA, Bongers PM, de Vroome EM. A new bricklayers' method for use in the construction industry. Scand J Work Environ Health. 2005; 31(5):394-400.
12. Blangsted AK, Søgaard K, Hansen EA, Hannerz H, Sjøgaard G. One-year randomized controlled trial with different physical-activity programs to reduce musculoskeletal symptoms in the neck and shoulders among office workers. Scand J Work Environ Health. 2008; 34(1):55-65.
13. von Thiele Schwarz U, Lindfors P, Lundberg U. Health-related effects of worksite interventions involving physical exercise and reduced workhours. Scand J Work Environ Health. 2008; 34(3):179-188.
14. Welch L, Haile E, Boden LI, Hunting KL. Musculoskeletal disorders among construction roofers - physical function and disability. Scand J Work Environ Health. 2009; 35(1):56-63.
15. Martimo KP, Shiri R, Miranda H, Ketola R, Varonen H, Viikari-Juntura E. Effectiveness of an ergonomic intervention on the productivity of workers with upper-extremity disorders--a randomized controlled trial. Scand J Work Environ Health. 2010; 36(1):25-33.
16. Driessen MT, Proper KI, Anema JR, Knol DL, Bongers PM, van der Beek AJ. The effectiveness of participatory ergonomics to prevent low-back and neck pain--results of a cluster randomized controlled trial. Scand J Work Environ Health. 2011; 37(5):383-393.
17. Jay K, Frisch D, Hansen K, Zebis MK, Andersen CH, Mortensen OS, et al. Kettlebell training for musculoskeletal and cardiovascular health: a randomized controlled trial. Scand J Work Environ Health. 2011; 37(3):196-203.
18. Shiri R, Martimo KP, Miranda H, Ketola R, Kaila-Kangas L, Liira H, et al. The effect of workplace intervention on pain and sickness absence caused by upper-extremity musculoskeletal disorders. Scand J Work Environ Health. 2011; 37(2):120-128.
19. Viikari-Juntura E, Kausto J, Shiri R, Kaila-Kangas L, Takala EP, Karppinen J, et al. Return to work after early part-time sick leave due to musculoskeletal disorders: a randomized controlled trial. Scand J Work Environ Health. 2012; 38(2):134-143.
20. Oude Hengel KM, Blatter BM, van der Molen HF, Bongers PM, van der Beek AJ. The effectiveness of a construction worksite prevention program on work ability, health, and sick leave: results from a cluster randomized controlled trial. Scand J Work Environ Health. 2013; 39(5):456-467.
21. Schoenfisch AL, Lipscomb HJ, Pompeii LA, Myers DJ, Dement JM. Musculoskeletal injuries among hospital patient care staff before and after implementation of patient lift and transfer equipment. Scand J Work Environ Health. 2013; 39(1):27-36.

22. Shiri R, Kausto J, Martimo KP, Kaila-Kangas L, Takala EP, Viikari-Juntura R. Health-related effects of early part-time sick leave due to musculoskeletal disorders: a randomized controlled trial. Scand J Work Environ Health. 2013; 39(1):37-45.
23. Vermeulen SJ, Heymans MW, Anema JR, Schellart AJM, van Mechelen W, van der Beek AJ. Economic evaluation of a participatory return-to-work intervention for temporary agency and unemployed workers sick-listed due to musculoskeletal disorders. Scand J Work Environ Health. 2013; 39(1):46-56.
24. Cantley LF, Taiwo OA, Galusha D, Barbour R, Slade MD, Tessier-Sherman B, et al. Effect of systematic ergonomic hazard identification and control implementation on musculoskeletal disorder and injury risk. Scand J Work Environ Health. 2014; 40(1):57-65.
25. Rantonen J, Vehtari A, Karppinen J, Luoto S, Viikari-Juntura E, Hupli M, et al. Face-to-face information combined with a booklet versus a booklet alone for treatment of mild low-back pain: a randomized controlled trial. Scand J Work Environ Health. 2014; 40(2):156-166.
26. Sundstrup E, Jakobsen MD, Brandt M, Jay K, Persson R, Aagaard P, et al. Workplace strength training prevents deterioration of work ability among workers with chronic pain and work disability: a randomized controlled trial. Scand J Work Environ Health. 2014; 40(3):244-251.
27. Jakobsen MD, Sundstrup E, Brandt M, Jay K, Aagaard P, Andersen LL. Effect of workplace- versus home-based physical exercise on musculoskeletal pain among healthcare workers: a cluster randomized controlled trial. Scand J Work Environ Health. 2015; 41(2):153-163.
28. Chaléat-Valayer E, Denis A, Abelin-Genevois K, Zelmar A, Siani-Trebern F, Touzet S, et al. Long-term effectiveness of an educational and physical intervention for preventing low-back pain recurrence: a randomized controlled trial. Scand J Work Environ Health. 2016; 42(6):510-519.
29. Danquah IH, Kloster S, Holtermann A, Aadahl M, Tolstrup JS. Effects on musculoskeletal pain from “Take a Stand!” – a cluster-randomized controlled trial reducing sitting time among office workers. Scand J Work Environ Health. 2017; 43(4):350-357.
30. Viikari-Juntura E, Virta LJ, Kausto J, Autti-Rämö I, Martimo KP, Laaksonen M, et al. Legislative change enabling use of early part-time sick leave enhanced return to work and work participation in Finland. Scand J Work Environ Health. 2017; 43(5):447-456.
31. Andersen LL, Geisle N, Knudsen B. Can beliefs about musculoskeletal pain and work be changed at the national level? Prospective evaluation of the Danish national Job & Body campaign. Scand J Work Environ Health. 2018; 44(1):25-36.
32. Jakobsen MD, Aust B, Kines P, Madeleine P, Andersen LL. Participatory organizational intervention for improved use of assistive devices in patient transfer: a single-blinded cluster randomized controlled trial. Scand J Work Environ Health. 2019; 45(2):146-157.
33. Leinonen T, Viikari-Juntura E, Husgafvel-Pursiainen K, Juvonen-Posti P, Laaksonen M, Solovieva S. The effectiveness of vocational rehabilitation on work participation: a propensity score matched analysis using nationwide register data. Scand J Work Environ Health. 2019; 45(6):651-660.
34. Pereira M, Comans T, Sjøgaard G, Straker L, Melloh M, O’Leary S, et al. The impact of workplace ergonomics and neck-specific exercise versus ergonomics and health promotion interventions on office worker productivity: A cluster-randomized trial. Scand J Work Environ Health 2019; 45 (1):42-52.
35. Viikari-Juntura E, Leinonen T, Virta LJ, Hiljanen I, Husgafvel-Pursiainen K, Autti-Rämö I, et al. Early part-time sick leave results in considerable savings in social security costs at national level: an analysis based on a quasi-experiment in Finland. Scand J Work Environ Health. 2019; 45(2):203-208.
36. Gismervik SØ, Aasdahl L, Vasseljen O, Fors EA, Rise MB, Johnsen R, et al. Inpatient multimodal occupational rehabilitation reduces sickness absence among individuals with musculoskeletal and common mental health disorders: a randomized clinical trial. Scand J Work Environ Health. 2020; 46(4):364-372.

37. Rasmussen CDN, Sørensen OH, van der Beek AJ, Holtermann A. The effect of training for a participatory ergonomic intervention on physical exertion and musculoskeletal pain among childcare workers (the TOY project) – a wait-list cluster-randomized controlled trial. Scand J Work Environ Health. 2020; 46(4):429-436.
38. Skagseth M, Fimland MS, Rise MB, Johnsen R, Borchgrevink PC, Aasdahl L. Effectiveness of adding a workplace intervention to an inpatient multimodal occupational rehabilitation program: A randomized clinical trial. Scand J Work Environ Health. 2020; 46(4):356-363.
39. Ryynänen K, Oura P, Simula AS, Holopainen R, Paukkunen M, Lausmaa M, et al. Effectiveness of training in guideline-oriented biopsychosocial management of low-back pain in occupational health services - a cluster randomized controlled trial. Scand J Work Environ Health. 2021; 47(5):367-376.
40. Waongenngarm P, van der Beek AJ, Akkarakittichoke N, Janwantanakul P. Effects of an active break and postural shift intervention on preventing neck and low-back pain among high-risk office workers: a 3-arm cluster-randomized controlled trial. Scand J Work Environ Health. 2021; 47(4):306-317.
